# Satisfaction with orthodontic treatment: cross-cultural adaptation and validation of an instrument for the Brazilian Portuguese language

**DOI:** 10.1590/2177-6709.27.6.e2220471.oar

**Published:** 2023-03-27

**Authors:** Renata Negreiros ALVARENGA, Saul Martins PAIVA, Carlos FLORES-MIR, Eduardo BERNABÉ, Lucas Guimarães ABREU

**Affiliations:** 1 Universidade Federal de Minas Gerais, Departamento de Saúde Bucal da Criança e do Adolescente (Belo Horizonte/MG, Brazil).; 2 University of Alberta, School of Dentistry (Edmonton/Alberta, Canada).; 3 Queen Mary University of London, Institute of Dentistry (London, United Kingdom).

**Keywords:** Orthodontics, Malocclusion, Patient satisfaction, Validation study, Surveys and questionnaires

## Abstract

**Objective:**

To cross-culturally adapt into the Brazilian Portuguese and evaluate the psychometric properties of an instrument for assessing the satisfaction of parents/guardians regarding their sons’/daughters’ orthodontic treatment.

**Methods:**

Translations of the instrument from English, pre-test and evaluation of validity and reliability of the Brazilian Portuguese version were performed. The questionnaire has 25 items distributed across 3 subscales (process, psychosocial effect and outcome). Eighty-three parents/guardians of children/adolescents who had completed orthodontic treatment participated. Descriptive statistics and floor and ceiling effects were calculated. Internal consistency, stability (interval of three weeks), convergent construct validity and discriminant construct validity were determined. Exploratory factor analysis (EFA) and confirmatory factor analysis (CFA) assessed dimensionality.

**Results:**

Among the 83 parents/guardians, 58 (69.9%) were mothers and 25 (30.1%) were fathers of children/adolescents. In the questionnaire’s total score and the three subscales scores, an acceptable percentage (≤15%) of participants achieved the maximum score (ceiling effect). In the total questionnaire score and in the three subscales scores, no participant achieved the minimum score (floor effect). Cronbach’s α coefficient for the total score was 0.72 (internal consistency). Intra-class correlation coefficient for the total score was 0.71 (stability). The questionnaire’s total score presented large Pearson correlation coefficient (>0.50) with the three subscales too (construct validity). Female parents/guardians had significantly higher scores in the psychosocial effect (*p*=0.013) and in the treatment outcome (*p*=0.037) subscales, compared to male parents/guardians (discriminant validity). EFA and CFA confirmed dimensionality in a three-factor solution.

**Conclusions:**

The final obtained version is valid and reliable to be used in Brazilian populations.

## RESUMO

**Objetivo:**

Adaptar transculturalmente para a língua portuguesa do Brasil e avaliar as propriedades psicométricas de um questionário que avalia a satisfação de pais e responsáveis com relação ao tratamento ortodôntico recebido por seus filhos.

**Métodos:**

Tradução do questionário em inglês, pré-teste e avaliação da validade e confiabilidade da versão em português obtida. O questionário consistiu de 25 itens distribuídos em três subescalas (processo durante o tratamento, efeito psicossocial e resultado geral do tratamento). Participaram 83 pais/responsáveis de crianças/adolescentes que concluíram o tratamento ortodôntico. Estatística descritiva foi realizada e foi calculado valor máximo e valor mínimo. Foram determinadas a consistência interna, a estabilidade (intervalo de três semanas), a validade de constructo convergente e a validade de constructo discriminante. A análise fatorial exploratória (AFE) e a análise fatorial confirmatória (AFC) avaliaram a dimensionalidade.

**Resultados:**

dos 83 pais/responsáveis que participaram do estudo, 58 (69,9%) eram mães e 25 (30,1%) eram pais das crianças/adolescentes. No escore total do questionário e no escore das três subescalas, uma porcentagem aceitável (≤15%) dos participantes atingiu o escore máximo (*ceiling effect*). No escore total do questionário e no escore das três subescalas, nenhum participante atingiu o escore mínimo (*floor effect*). O coeficiente α de Cronbach para o escore total foi 0,72 (consistência interna). O coeficiente de correlação intraclasse para o escore total foi 0,71 (estabilidade). O escore total do questionário apresentou um coeficiente de correlação de Person alto (>0,50) com as três subescalas (validade de constructo). Pais/responsáveis do sexo feminino apresentaram escores significativamente maiores nas subescalas efeito psicossocial (*p*=0,013) e desfecho do tratamento (*p*=0,037), em comparação aos pais/responsáveis do sexo masculino (validade discriminante). AFE e AFC confirmaram a dimensionalidade em uma solução de três fatores.

**Conclusões:**

A versão do questionário obtida no presente estudo mostrou-se válida e confiável para o uso na população brasileira.

## INTRODUCTION

Interest in patient satisfaction with health care has grown in recent years.^1^ Patients’ perceptions and expectations have become increasingly important to justify the provision of healthcare services and guarantee its general quality.^2^ Measuring the satisfaction associated with orthodontic treatment process is complex, as multiple dimensions of treatment must be considered simultaneously.^1^ Generally, the level of satisfaction with orthodontic treatment is assessed by the individual’s perception of the final alignment and leveling of his/her teeth or only by the result of the treatment itself, being assessed through simple questionnaires or questionnaires developed for general dental practice. However, the result of orthodontic treatment does not involve just aligning and leveling the teeth or having good occlusion. Therefore, there is a need for a more comprehensive questionnaire that provides data that allow clinicians and orthodontic care service organizers to reflect on the specific level of satisfaction with the orthodontic treatment.^3^

In a systematic review carried out in 2015, several factors associated with satisfaction in orthodontic treatment of patients and their guardians after completion of treatment were identified. In general, satisfaction was associated with pleasant aesthetic results perceived by patients, perception of psychological benefits with the treatment and good quality of care related to the interactions of patients with the orthodontist and his/her team.^4^ However, in most studies, the assessment of satisfaction with orthodontic treatment was performed with surveys with a limited number of questions, whose psychometric properties had not been validated. Moreover, in the Brazilian Portuguese language, there is no validated questionnaire that addresses orthodontic outcomes.^4^

Bennett et al.^1^ developed a questionnaire in the English language that addresses aspects related to the satisfaction of parents/guardians of children/adolescents undergoing orthodontic treatment. Given the lack of a questionnaire in Brazilian-Portuguese that is a reliable instrument for assessing parents’/caregivers’ satisfaction with the orthodontic treatment of their children/adolescents, the aim of this study was to cross-culturally adapt into the Brazilian Portuguese and evaluate the psychometric properties of the adapted version of the questionnaire developed by Bennett et al.^1^

## METHODS

### ETHICAL CONSIDERATIONS

The Ethics Committee of the Federal University of Minas Gerais (Brazil) approved this study (06898519.4.0000.5149).

### INSTRUMENT DESCRIPTION

The original questionnaire in English language is a specific condition instrument developed in North Carolina, United States, created to assess the satisfaction of parents/guardians of individuals under 18 years of age who had undergone orthodontic treatment. This instrument consists of 25 questions distributed across 3 subscales: satisfaction with the treatment process (13 items), psychosocial effect of the treatment (7 items) and treatment outcome (5 items). Each item has 5 response options, according to the Likert scale, ranging from 1 to 5 (1=strongly disagree, 2=disagree, 3=neither agree nor disagree, 4=agree and 5=strongly agree). The scores for the response of items 11 and 25 should be reversed (Appendix 1 [Fig f1a]). The questionnaire’s total score ranges between 25 and 125. The higher the score, the greater the satisfaction of the parent/guardian with the child’s/adolescent’s orthodontic treatment. The scores of the subscales range as follows: treatment process (13 - 65), psychosocial effect of treatment (7 - 35) and treatment outcome (5 - 25). The higher the score, the greater the satisfaction of the parent/guardian with respect to the construct assessed in the subscales.^1^

### TRANSLATION AND CROSS-CULTURAL ADAPTATION OF THE INSTRUMENT

The stages of this study followed international standards for translation, cross-cultural adaptation and validation of instruments^5^ for the assessment of health outcomes.

First, the instrument was translated from English into Brazilian Portuguese by two different independent professionals, who were native in Brazilian Portuguese, fluent in English and with knowledge in Dentistry and Orthodontics. In order to preserve the concept and the equivalence of the instrument’s items, the two translated versions were evaluated by a multidisciplinary committee, with all members native in Brazilian Portuguese, with knowledge in Dentistry and Orthodontics and also fluent in English. The objective of this committee was to identify any inconsistencies in translation that could cause difficulties for any native speaker in understanding any of the questions, and to synthesize a single Brazilian Portuguese version of the instrument.

This first Brazilian Portuguese version of the instrument was then back-translated into the original English language by an individual native to the English language and fluent in Brazilian Portuguese, not involved in the first translation phase. The translator also did not have access to the original English instrument. After performing the back-translation, this English version produced by the back-translator was sent to the authors of the original instrument who did not suggest any modifications in the back-translated questionnaire. After all this process, a version of the instrument in the Brazilian Portuguese language was set (Appendix 2 [Fig f2a]).

**Figure 1:**
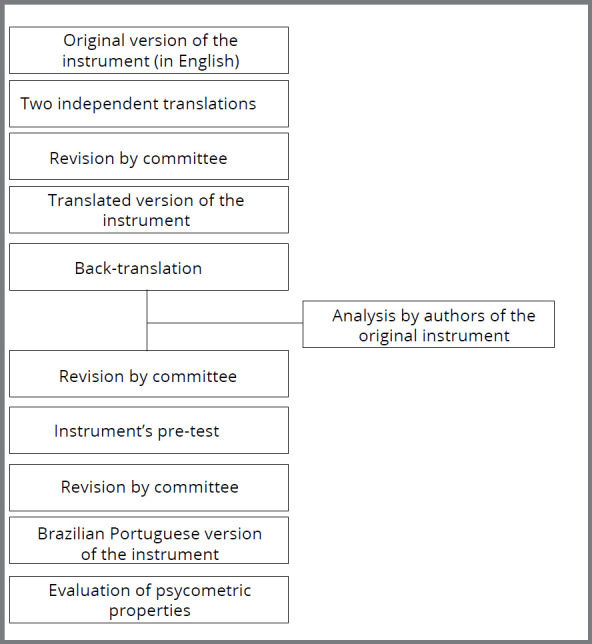
Flowchart illustrating the complete process of translation, cross-cultural adaptation and validation of the instrument.

After reaching a Brazilian Portuguese version, a pre-test was performed with a convenience sample of 15 parents/guardians of individuals under 18 years of age who had completed orthodontic treatment. Pre-test participants were not included in the main study. The purpose was to find possible difficulties in understanding the instrument by laypersons without a background in oral health practice. They were encouraged to suggest any synonyms for terms or words that were difficult to understand. A flowchart illustrating the complete process of translation, cross-cultural adaptation and validation of the instrument is provided in Figure 1.

### EVALUATION OF THE VALIDITY AND RELIABILITY OF THE BRAZILIAN PORTUGUESE VERSION

The main sample of this study comprised 83 parents/guardians of children/adolescents under the age of 18 who had completed orthodontic treatment in two orthodontic clinics. As inclusion criteria, these individuals had to be native Brazilian Portuguese speakers. Children/adolescents could not have craniofacial anomalies or cognitive disorders.

Parents/guardians filled out a clinical form where the following information was collected: name of the child/adolescent and their parents/guardian; child’s/adolescent’s sex; child’s/adolescent’s date of birth and age; family income (number of minimum wages earned by all family members who were economically active); number of people who lived from this income; number of children/adolescents in the household; parents’/guardian level of education (number of years of education); which orthodontics device was worn (type of treatment received by the children/adolescents) and the initial condition of malocclusion of children/adolescents, assessed by examining initial plaster models (before treatment) with the Dental Aesthetic Index (DAI). In DAI, 10 items were assessed: number of incisors, canines and pre-molars missing, crowding and spacing in the incisors area, diastema between maxillary central incisors, greatest irregularity in the maxillary incisors, greatest irregularity in the mandibular incisors, maxillary overjet, mandibular overjet, open bite and sagittal relationship of molars. Based on the cutoff points of the DAI, children/adolescents were classified into four severity levels of malocclusion, with different recommendations for orthodontic treatment: mild malocclusion/slight need for treatment (DAI≤25), defined malocclusion/elective treatment (26≤DAI≤30), severe malocclusion/highly desirable treatment (31≤DAI≤35), and very severe malocclusion/mandatory treatment (DAI≥36).^6^ Family income was measured by using the Brazilian minimum wage as reference (US$ 200) on the date of data collection, and was categorized as ≤2 minimum wages, =3 minimum wages or ≥4 minimum wages.

Then, the final Brazilian version of the questionnaire was self-applied, and the 83 parents/guardians of children/adolescents answered the questionnaire in a separate room with a researcher available to answer any questions. Parents/caregivers answered the questionnaire twice, with an interval of three weeks to verify stability (test-retest). The same researcher collected data in the test and in the retest. 

### STATISTICAL ANALYSIS

Statistical analysis was conducted using the Statistical Package for the Social Sciences software (SPSS for Windows, v. 23.0, IBM, Armonk, USA) and the Amos software (Amos for Windows, v. 26.0, IBM, Armonk, USA). A descriptive analysis with the sociodemographic characteristics of the sample, the severity of the malocclusion and type of orthodontic treatment to which the children/adolescent had been submitted was performed. 

Data on the total questionnaire’s score and on the subscales’ scores presented normal distribution. The convergent construct validity was assessed using Pearson’s coefficient, through the correlation between the subscale scores and the total questionnaire score. Pearson’s coefficient is interpreted as follows: <0.30 (small), 0.30-0.50 (average) and >0.50 (large).^7^ The discriminant construct validity was determined by comparing male and female^8^ parents/guardians who had answered the questionnaire, in relation to the subscale scores and the total questionnaire score. The Student *t*-test was used.

The reliability of the instrument was examined by assessing internal consistency and test-retest stability. Internal consistency was assessed using the Cronbach α coefficient. Values greater than or equal to 0.70 are considered acceptable.^9^ Test-retest stability was determined using the intraclass correlation coefficient (ICC). ICC values are interpreted as follows: ICC < 0.20 (weak correlation), ICC = 0.20-0.40 (fair correlation), ICC = 0.41-0.60 (moderate correlation), ICC = 0.61-0.80 (good correlation) and ICC = 0.8 -1.00 (excellent correlation).^10^

The mean and standard deviation of the subscale scores, the total questionnaire score and the percentage of individuals obtaining the maximum value (ceiling effect) and the minimum value (floor effect) of the subscale scores and the total questionnaire score were also determined. The ideal maximum percentage for both maximum and minimum values is 15%.^11^

Exploratory factor analysis (EFA) was carried out to assess the questionnaire’s dimensionality. Data set suitability was checked employing the Barlett’s test of sphericity (p<0.05) and the Kayser-Meyer-Olkin (KMO) measure (>0.50). The factors were extracted with the principal components analysis. The basis for the determination of the number of factors were the method deployed in the development of the questionnaire in English and the screen plot assessment. Rotation was performed with the Promax method. In the matrix, the items with factor loadings >0.40 were clustered together. Confirmatory factor analysis (CFA) was performed to ratify the dimensionality of the questionnaire. The Comparative Fit Index (CFI) was determined, and a value >0.90 denotes a satisfactory model fit.^12^ ^,^ ^13^

## RESULTS

Among the 83 parents/guardians who participated, 58 were women and 25 were men. Children’s/adolescents’ mean age was 13.0 years (±3.07) - 41 were boys and 42 were girls. Sample’s sociodemographic characteristics, malocclusion severity and the type of orthodontic treatment received by children/adolescents are shown in Table 1.

**Table 1:** Sociodemographic characteristics of the sample, severity of malocclusion and type of orthodontic treatment received by children/adolescents.

	n (%)
SEX OF PARENTS/GUARDIANS	
Male	25 (30.1)
Female	58 (69.9)
SEX OF CHILDREN/ADOLESCENTS	
Male	41 (49.4)
Female	42 (50.6)
FAMILY INCOME (BASED ON THE MINIMUM WAGE)	
≤2 minimum wages	31 (37.3)
=3 minimum wages	25 (30.2)
≥4 minimum wages	27 (32.5)
NUMBER OF PEOPLE WHO LIVE FROM THIS INCOME	
≤3 people	32 (38.6)
>3 people	51 (61.4)
NUMBER OF CHILDREN/ADOLESCENTS IN THE HOUSEHOLD	
=1 child	20 (24.1)
=2 children	45 (54.2)
≥3 children	18 (21.7)
PARENTS’/GUARDIAN’S SCHOOLING	
≤9 years of education	33 (39.8)
>9 years of education	50 (60.2)
CHILDREN’S/ADOLESCENTS’ DAI (BEFORE TREATMENT)	
≤25 (mild malocclusion)	11 (13.3)
=26-30 (defined malocclusion)	25 (30.1)
=31-35 (severe malocclusion)	22 (26.5)
≥36 (very severe malocclusion)	25 (30.1)
TREATMENT RECEIVED BY THE CHILDREN/ADOLESCENTS	
Interceptive	33 (39.7)
Corrective	32 (38.6)
Interceptive and Corrective	18 (21.7)

Minimum wage at the time of data collection was US$ 200.00. DAI = Dental Aesthetic Index.

For the convergent construct validity, the total score of the questionnaire reached a high Pearson correlation coefficient (> 0.50) within the three subscales. The values of the construct validity (Pearson’s correlation) are shown in Table 2. For the discriminant construct validity, female parents/guardians had significantly higher scores for the psychosocial effect (p=0.013) and treatment outcome (p=0.037) subscales compared to male parents/guardians (Table 3).

**Table 2:** Construct validity. Pearson’s correlation.

	Treatment process	Psychosocial effect of treatment	Treatment outcome	Total questionnaire score
Treatment process	1	0.41*	0.19	0.87*
Psychosocial effect of treatment		1	0.37*	0.76*
Treatment outcome			1	0.51*
Total questionnaire score				1

*p<0.001.

**Table 3:** Discriminant validity. Comparison between male and female parents/guardians with respect to the orthodontic treatment of their sons/daughters.

	Sex of parents/guardians who answered the questionnaire		p value*
	Male Mean (SD)	Female Mean (SD)	
Treatment process	56.88 (5.46)	57.17 (5.28)	0.819
Psychosocial effect of treatment	26.96 (3.97)	29.40 (4.02)	0.013
Treatment outcome	21.04 (2.63)	22.16 (1.98)	0.037
Total questionnaire score	104.88 (9.97)	108.72 (8.89)	0.085

SD=standard deviation, *Student t-test. Significant at p<0.05.

The higher the mean score, the greater the satisfaction of the parents/guardians with the children’s/adolescents’ orthodontic treatment.

Regarding internal consistency, Cronbach’s α coefficient value for the total score of the questionnaire was of 0.72. For the subscales, values ranged from 0.68 (treatment outcome) to 0.75 (treatment process). Regarding test-retest reliability, the ICC value for the total score of the questionnaire was 0.71, indicating a good correlation. For the subscales, the values ranged from 0.68 (psychosocial effect of treatment and treatment outcome) to 0.76 (treatment process). A percentage of individuals slightly higher than 15% reaching the maximum score was only found in the treatment outcome subscale. In the questionnaire’s total score and in the three subscales, the percentage of individuals reaching the minimum score was of 0% (Table 4).

**Table 4:** Descriptive analysis and reliability of the questionnaire assessing satisfaction of parents/guardians with respect to the orthodontic treatment of their sons/daughters.

	Number of items	Score range	Mean (SD)	Ceiling effect %	Floor effect %	Cronbach α	ICC
Treatment process	13	13 - 65	57.28 (6.44)	4.8	0	0.75	0.76
Psychosocial effect of treatment	7	7 - 35	29.46 (3.99)	2.4	0	0.69	0.68
Treatment outcome	5	5 - 25	22.08 (2.25)	15.6	0	0.68	0.68
Total questionnaire score	25	25 - 125	108.82 (9.82)	1.2	0	0.72	0.71

SD=standard deviation, ICC=intra-class correlation coefficient.

**Figure 2:**
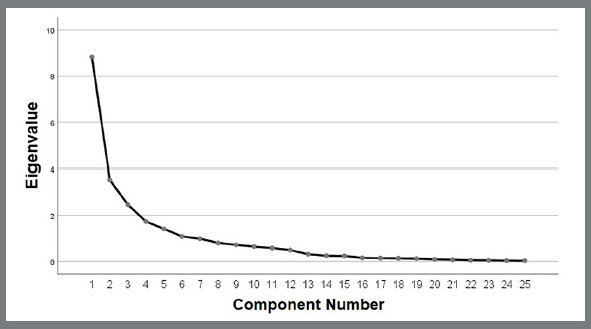
Scree plot showing the relationship between the component numbers and the eigenvalues.

**Figure 3:**
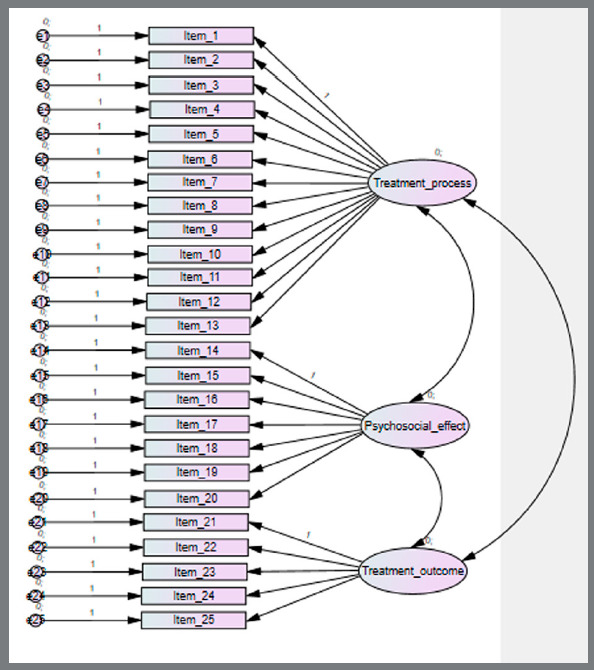
Model of the Confirmatory Factor Analysis.

The value of KMO=0.743 and the significance of the Barlett’s test of sphericity (p<0.001) confirmed the feasibility of the EFA. A graph displaying the relationship between the component numbers and the eigenvalues is showed in Figure 2. The three-factor solution explained 59.35% of the overall variance. Factor I consisted of 12 items, accounting for 35.34% of the variance. Factor II comprised six items, accounting for 14.15% of the variance. Factor III was composed of seven items, accounting for 9.86% of the variance. Cronbach’s coefficients for Factor I, Factor II and Factor III were 0.91, 0.89 and 0.71 (above the level recommended). The clustering of the items in the three-factor solution was quite similar to the original instrument (Table 5). Figure 3 illustrates the model of the CFA. Most items demonstrated high factor loadings. Only four items (1, 4, 21 and 25) had low factor loadings (<0.40). Four items had factor loadings ranging between 0.40 and 0.50 (Table 5). The CFA of the structure was also tested, with items 4 and 14 fitting in Factor III (CFA 2), and the results were very much alike to the previous CFA test. Only three items (1, 21 and 25) had low factor loadings (<0.40). To enhance the model fit, error variance was added, and the value of CFI was >0.90, indicating appropriate goodness of fit. 

**Table 5:** Factor loadings for the three-factor EFA and for the CFA of the questionnaire assessing satisfaction of parents/guardians regarding the orthodontic treatment of their sons/daughters.

	EFA			Error variance		CFA
	Factor I	Factor II	Factor III	Estimate	SE	
Subscale 1						
Item 1	0.573	-0.367	0.004	0.285	0.045	0.323
Item 2	0.936	-0.101	-0.250	0.116	0.020	0.805
Item 3	0.897	0.005	-0.211	0.176	0.030	0.814
Item 4	0.165	-0.258	0.680	0.870	0.136	0.240
Item 5	0.848	0.087	0.021	0.061	0.011	0.893
Item 6	0.868	-0.064	0.178	0.056	0.011	0.909
Item 7	0.894	0.001	0.013	0.073	0.015	0.928
Item 8	0.730	0.063	0.211	0.066	0.011	0.791
Item 9	0.520	0.114	0.027	0.371	0.059	0.477
Item 10	0.735	0.147	-0.196	0.406	0.066	0.692
Item 11	0.489	-0.144	0.107	0.922	0.145	0.420
Item 12	0.823	-0.011	0.166	0.219	0.039	0.861
Item 13	0.529	0.377	-0.082	0.284	0.045	0.626
Subscale 2						
Item 14	0.199	0.265	0.481	0.414	0.068	0.585
Item 15	0.103	0.710	0.298	0.125	0.026	0.855
Item 16	-0.084	0.732	0.187	0.231	0.040	0.738
Item 17	0.039	0.850	0.063	0.112	0.024	0.867
Item 18	-0.011	0.853	-0.124	0.174	0.031	0.759
Item 19	0.005	0.878	-0.341	0.311	0.052	0.652
Item 20	-0.140	0.874	-0.055	0.379	0.066	0.722
Subscale 3						
Item 21	-0.282	0.164	0.463	0.875	0.140	0.321
Item 22	0.015	-0.087	0.839	0.126	0.030	0.745
Item 23	-0.037	0.317	0.592	0.099	0.035	0.827
Item 24	0.150	0.096	0.437	0.290	0.048	0.467
Item 25	-0.155	-0.199	0.682	0.644	0.101	0.195

Subscale 1=treatment process, Subscale 2=psychosocial effect, Subscale 3=treatment outcome.

EFA=exploratory factor analysis, SE=standard error, CFA=confirmatory factor analysis.

BOLD INDICATES FACTOR LOADINGS >0.40.

## DISCUSSION

Confirmation of the convergent construct validity is given when two instruments that assesses the same construct have a strong positive correlation.^14^ Herein, this confirmation could have been achieved by correlating the validated questionnaire in this study with another questionnaire that evaluated a construct similar regarding the satisfaction of parents/guardians with the orthodontic treatment of their sons/daughters. However, this assessment was made by correlating the total score of the questionnaire with the subscales of the questionnaire itself. Since subscales and the total score of the questionnaire evaluate the same construct (satisfaction), it would be expected that the correlations were greater than 0.50,^15^ which was confirmed for the three subscales.

The evaluation of the discriminant construct validity is a very important and useful psychometric property for detecting differences between two groups of recognizably different individuals.^16^ In the present study, the discriminant validity was determined by comparing male and female parents/guardians who had answered the questionnaire, in relation to the subscale scores and the instrument’s total score. The literature acknowledges the influence of the variable sex in relation to the satisfaction of individuals with health services, showing significant differences between female and male individuals.^17^ ^,^ ^18^ The results of the present study showed that female parents/guardians had significantly higher scores for the psychosocial effect and treatment outcome subscales, compared to male parents/caregivers, indicating greater mothers’ satisfaction in regards to orthodontic treatment of their sons/daughters for these subscales. To date, however, the related literature has suggested otherwise. Compared to men, women have a less positive perception of satisfaction with the health service offered to them and less enthusiasm for the treatment received.^8^ The present results may be related to the fact that mothers are the primary decision makers regarding their sons’/daughters’ health.^19^ The involvement of female parents/guardians may have increased the levels of satisfaction, leading to a more positive perception of mothers towards children’s/adolescents’ orthodontic treatment.

The internal consistency, determined by the Cronbach α coefficient, assesses the extent to which the subscale items and all items in the questionnaire assess the same construct. High values of this coefficient show that the items within the subscales and the items that make up the total score of the questionnaire supposed to be evaluating the same construct are correlating well.^9^ The total score of the questionnaire showed a Cronbach’s α coefficient greater than 0.70. For the subscales, the values were close to this limit for acceptability, with only two subscales with values slightly lower than the threshold. In the study for the development of the original instrument in English, Cronbach’s α coefficients were also greater than 0.70. It is noteworthy that this coefficient is strongly influenced by the number of items in the subscale and the sample size.^20^ However, even if we consider the cut-off of acceptability as a rule of thumb, a slightly diminished Cronbach α coefficient does not necessarily imply that the questionnaire is unsatisfactory.^21^

The reliability of an instrument was ratified by the assessment of stability and internal consistency. To assess stability (test-retest), the instrument was answered by parents/guardians twice with a 21-day interval. Instruments for assessing health outcomes should be reproducible over time,^22^ that is, the results obtained from the responses of parents/guardians should be similar at both times, since the clinical status of children/adolescents had not changed in one short interval of three weeks. In the present study, all the 83 parents/guardians answered the questionnaire twice. ICC results demonstrated that the stability of the instrument over time was adequate. Neither the total score nor the three subscales exhibited excellent ICC values. However, the values were within the range that indicates good correlation, being satisfactory in studies assessing the psychometric properties of questionnaires.^23^ The study for the development of the original instrument also demonstrated that the questionnaire is reliable to assess the three dimensions of the satisfaction of parents/guardians regarding the orthodontic treatment of their sons/daughters.^1^ During any study of cross-cultural adaptation and validation of a survey, an adequate definition of this interval between the two applications of the questionnaire is important since it must be long enough to minimize the effects of memory bias and, at the same time, short so that the assessed condition of the patient evaluated does not change.^14^

The minimum value (floor effect) is a limitation that occurs when the lowest score of the questionnaire that can be obtained is reached by a significant number of individuals, decreasing the probability that the tested instrument has accurately measured the subscale or the construct that is being assessed. Thus, if a large percentage of individuals reaches the minimum value of a subscale or the total score of a questionnaire, the question whether the study participants really read, understood, and filled the questionnaire correctly remains unanswered.^24^ In the present study, the percentage of individuals reaching the minimum value of the subscales and the total score of the questionnaire was 0%, which was much lower than the set limit percentage of 15%. For the maximum value (ceiling effect), results were also satisfactory. A very large percentage of maximum or minimum values could have undermined the validity of the questionnaire, resulting in impaired future cross-sectional assessments, due to the lack of accuracy of the instrument to demonstrate the individual’s condition at that moment. Longitudinal assessments may also be jeopardized, as the instrument would be unable to detect changes in the patient’s clinical status over time. Excessive percentages of maximum and minimum values are also an indication that there is lack of options at the maximum or minimum end of the response scale, denoting a deficiency in the instrument’s content validity.^11^ ^,^ ^22^

In studies of validation of questionnaires, EFA is employed to verify the connection that exists between the variable assessed and the individuals who responded the questionnaire. Usually, oblique rotation rather than orthogonal rotation is used for this purpose. It is reasonable to test the solutions provided by different types of oblique rotations.^25^ In the present study, the promax rotation produced the most adequate solution and, thus, was used as the basis of the interpretation. Regarding the number of factors extracted, the literature has recommended that the number of eigenvalues higher than one is helpful in determining the number of factors retained.^25^ ^,^ ^26^ Herein, the study in which the original instrument was developed^1^ and the visual assessment of the scree plot depicting the relationship between the component numbers and the eigenvalues determined the number of factors extracted. EFA should be confirmed by CFA, ratifying the theory underlying the structure of the phenomena evaluated.^27^ In the present study, the value of CFI indicated that the data observed fitted the theoretical model.

The final methodological issue that deserves a discussion is the use of DAI for malocclusion assessment, rather than other available indices. DAI aggregates aesthetic and clinical characteristics numerically to provide a unique score that can be analyzed as a continuous or a categorical variable.^6^ ^,^ ^28^ In comparison with alternative indices, DAI is easier to use and time saving during data collection.^28^

This instrument has several potential uses related to the satisfaction of parents/guardians regarding the orthodontic treatment of their sons/daughters. First, it allows orthodontists to reliably assess which factors are responsible for the satisfaction or dissatisfaction of individuals with offered services, thus being able to adapt their conduct and provide care centered on their patient. Patient-centered care can be defined as providing respectful and responsive care to child/adolescent patients’ and their parents’/guardians’ individual preferences, needs and values, and ensuring that these values guide all clinical decisions.^29^ ^,^ ^30^ During treatment, patient-centered care increases the satisfaction of patients and their parents/guardians alike, therefore increasing their adherence to treatment, bringing better final results. Another point concerns the orthodontist’s support team and his/her service area. The instrument allows the clinician to assess how the parent’s satisfaction with these two aspects is and direct the professional’s attention to a more humanized service. It is safe to say that improving the quality of health services has become crucial for the operational aspects of health centers.^30^ ^,^ ^31^

Future evaluations must be carried out to confirm the instrument’s psychometric properties in studies with a population different from the population of the city where the instrument was validated, allowing researchers to obtain more accurate estimates. Further longitudinal studies^32^ will provide a better understanding of the factors related to the satisfaction of parents/ guardians with the orthodontic treatment of their children/adolescents, allowing orthodontists to have a better understanding of such factors, directing their attention to patient care. Prospective studies evaluating the participants before and after an orthodontic intervention will also allow the assessment of other psychometric properties, such as responsiveness and the minimal important clinical difference, impossible to be tested in the present study without such evaluations.^33^ Finally, this instrument can fulfill an important role for Orthodontics, since in Brazil, orthodontists still use instruments whose properties have not yet been tested or instruments designed to evaluate the general practice, often inappropriate for use in the context of a specialty.^34^

## CONCLUSION

The total score and the three subscales scores of the modified instrument demonstrated adequate psychometric properties. The results of this study show that this instrument is reliable for being applied in Brazilian parents/guardians of children/adolescents who have completed orthodontic treatment.

## References

[1] Bennett ME, Tulloch JF, Vig KW, Phillips CL (2001). Measuring orthodontic treatment satisfaction questionnaire development and preliminary validation. J Public Health Dent.

[2] Bailey LJ, Duong HL, Proffit WR (1998). Surgical Class III treatment long-term stability and patient perceptions of treatment outcome. Int J Adult Orthodon Orthognath Surg.

[3] Lee R, Hwang S, Lim H, Cha JY, Kim KH, Chung CJ (2018). Treatment satisfaction and its influencing factors among adult orthodontic patients. Am J Orthod Dentofacial Orthop.

[4] Pachêco-Pereira C, Pereira JR, Dick BD, Perez A, Flores-Mir C (2015). Factors associated with patient and parent satisfaction after orthodontic treatment a systematic review. Am J Orthod Dentofacial Orthop.

[5] Paiva SM, Firmino RT, Abreu LG, Estrela C (2018). Metodologia científica: ciência, ensino, pesquisa.

[6] Jenny J, Cons NC (1996). Establishing malocclusion severity levels on the Dental Aesthetic Index (DAI) scale. Aust Dent J.

[7] Cohen J (1988). Statistical power analysis for the behavioral sciences.

[8] Crow R, Gage H, Hampson S, Hart J, Kimber A, Storey L (2002). The measurement of satisfaction with healthcare implications for practice from a systematic review of the literature. Health Technol Assess.

[9] Cronbach LJ (1951). Coefficient alpha and the internal structure of tests. Psychometrika.

[10] Landis JR, Koch GG (1977). The measurement of observer agreement for categorical data. Biometrics.

[11] Terwee CB, Bot SD, de Boer MR, van der Windt DA, Knol DL, Dekker J (2007). Quality criteria were proposed for measurement properties of health status questionnaires. J Clin Epidemiol.

[12] Bentler PM (1990). Comparative fit indexes in structural models. Psychol Bull.

[13] Kline RB (2015). Principles and practice of structural equation modeling.

[14] Kimberlin CL, Winterstein AG (2008). Validity and reliability of measurement instruments used in research. Am J Health Syst Pharm.

[15] Strauss ME, Smith GT (2009). Construct validity advances in theory and methodology. Annu Rev Clin Psychol.

[16] Polit DF (2015). Assessing measurement in health beyond reliability and validity. Int J Nurs Stud.

[17] Weisman CS, Rich DE, Rogers J, Crawford KG, Grayson CE, Henderson JT (2000). Gender and patient satisfaction with primary care tuning in to women in quality measurement. J Womens Health Gend Based Med.

[18] Woods SE, Heidari Z (2003). The influence of gender on patient satisfaction. J Gend Specif Med.

[19] Boland L, Kryworuchko J, Saarimak A, Lawson ML (2017). Parental decision making involvement and decisional conflict a descriptive study. BMC Pediatr.

[20] Aaronson N, Alonso J, Burnam A, Lohr KN, Patrick DL, Perrin E (2002). Assessing health status and quality-of-life instruments attributes and review criteria. Qual Life Res.

[21] Hair JF, Money AH, Page M, Samouel P (2007). Research methods for business.

[22] Keszei AP, Novak M, Streiner DL (2010). Introduction to health measurement scales. J Psychosom Res.

[23] Bartko JJ (1966). The intraclass correlation coefficient as a measure of reliability. Psychol Rep.

[24] McHorney CA, Tarlov AR (1995). Individual-patient monitoring in clinical practice are available health status surveys adequate?. Qual Life Res.

[25] Gaskin CJ, Happell B (2014). On exploratory factor analysis a review of recent evidence, an assessment of current practice, and recommendations for future use. Int J Nurs Stud.

[26] Larsen R, Warne RT (2010). Estimating confidence intervals for eigenvalues in exploratory factor analysis. Behav Res Methods.

[27] Marsh HW, Guo J, Dicke T, Parker PD, Craven RG (2020). Confirmatory Factor Analysis (CFA), Exploratory Structural Equation Modeling (ESEM), and Set-ESEM optimal balance between goodness of fit and parsimony. Multivariate Behav Res.

[28] Jenny J, Cons NC (1996). Comparing and contrasting two orthodontic indices, the Index of Orthodontic Treatment need and the Dental Aesthetic Index. Am J Orthod Dentofacial Orthop.

[29] Kuipers SJ, Cramm JM, Nieboer AP (2019). The importance of patient-centered care and co-creation of care for satisfaction with care and physical and social well-being of patients with multi-morbidity in the primary care setting. BMC Health Serv Res.

[30] Tas FV, Guvenir T, Cevrim E (2010). Patients' and their parents' satisfaction levels about the treatment in a child and adolescent mental health inpatient unit. J Psychiatr Ment Health Nurs.

[31] Chang WJ, Chang YH (2013). Patient satisfaction analysis Identifying key drivers and enhancing service quality of dental care. J Dent Sci.

[32] Caruana EJ, Roman M, Hernández-Sánchez J, Solli P (2015). Longitudinal studies. J Thorac Dis.

[33] Revicki DA, Cella D, Hays RD, Sloan JA, Lenderking WR, Aaronson NK (2006). Responsiveness and minimal important differences for patient reported outcomes. Health Qual Life Outcomes.

[34] Oliveira PG, Tavares RR, Freitas JC (2013). Assessment of motivation, expectations and satisfaction of adult patients submitted to orthodontic treatment. Dental Press J Orthod.

